# Relay Positioning for Load-Balancing and Throughput Enhancement in Dual-Hop Relay Networks

**DOI:** 10.3390/s21051914

**Published:** 2021-03-09

**Authors:** Byungkwan Kim, Taejoon Kim

**Affiliations:** School of Information and Communication Engineering, Chungbuk National University, Chungju 28644, Korea; bking@chungbuk.ac.kr

**Keywords:** dual-hop, relay location, load-balancing, throughput enhancement

## Abstract

In a cellular communication system, deploying a relay station (RS) is an effective alternative to installing a new base station (BS). A dual-hop network enhances the throughput of mobile stations (MSs) located in shadow areas or at cell edges by installing RSs between BSs and MSs. Because additional radio resources should be allocated to the wireless link between BS and RS, a frame to be transmitted from BS is divided into an access zone (AZ) and a relay zone (RZ). BS and RS communicate with each other through the RZ, and they communicate with their registered MSs through an AZ. However, if too many MSs are registered with a certain BS or RS, MS overloading may cause performance degradation. To prevent such performance degradation, it is very important to find the proper positions for RSs to be deployed. In this paper, we propose a method for finding the sub-optimal RS deployment location for the purpose of load-balancing and throughput enhancement. The advantage of the proposed method is the efficiency in find the sub-optimal location of RSs and its reliable tradeoff between load-balancing throughput enhancement. Since the proposed scheme finds the proper position by adjusting the distance and angle of RSs, its computational complexity lower than other global optimization approach or learning-based approach. In addition, the proposed scheme is constituted with the two stages of load-balancing and throughput enhancement. These procedures result in the appropriate tradeoff between load-balancing and throughput enhancement. The simulation results support these advancements of the proposed scheme.

## 1. Introduction

Multi-hop relay systems have been studied as a key technology to increase the transmission rate and reliability in cellular communication systems, with a low infrastructure cost [1,2,3]. The multi-hop relay system improves the channel quality between a base station (BS) and mobile stations (MSs) by deploying a relay station (RS) between the existing BS and MSs. RS may operate in either transparent or non-transparent mode [4]. When an RS operates in transparent mode, the donor BS and the RS transmit the same signal to MSs simultaneously to increase the channel capacity, and the MSs do not recognize the existence of the RS. Meanwhile, when an RS operates in the non-transparent mode, it decodes the signal received from a donor BS and transmits the re-encoded signal to MSs, which eliminates shadow areas caused by signal attenuation. Moreover, since RSs can be installed at a lower infrastructure cost than BSs, the cell coverage of a conventional single-hop system can be extended more economically and efficiently.

In a multi-hop relay system, additional wireless resource should be allocated to the link between a donor BS and RSs. In setting aside the required resource for the communication between a donor BS and RSs, a time-division duplex (TDD) or a frequency-division duplex (FDD) scheme is applied to minimize the interference between the BS and RSs [5]. Because whether an MS is registered to a BS or an RS determines wireless resource to be used for the MS, it is important for the MS to select an appropriate service node, i.e., BS or RS, to which it registers. If a certain BS or an RS is overloaded with many MSs, a resource shortage is inevitable, and the radio resource of other BSs or RSs would be under-used resulting in degradation of the system performance.

To prevent such performance degradation by overloading, methods of switching the service node of MSs were proposed, considering the traffic load condition [6,7]. However, these schemes do not guarantee the best channel quality for the MSs after switching the service nodes. Moreover, the scheme in [6] cannot completely resolve the performance degradation because the service node of MSs changes after the overload has already occurred. Moreover, once installed, it is rather costly to move RSs. In [8,9], the authors proposed a method of increasing the amount of resource to be allocated to BS or RSs by adopting a frequency reuse scheme when resource shortage happens due to overloading. However, reusing the frequency resources may cause severe intracell interference. Because the above-mentioned schemes also commonly increase system complexity, it is important to minimize the possibility of overloading at the design level of a relay system.

The signal to interference plus noise ratio (SINR) distribution of MSs and the shadow areas in a cell are greatly affected by the RSs’ position. Accordingly, the installation location of each RS is a very important parameter determining the overall performance of a relay system. In [10], a heuristic algorithm to determine the number and location of RS was studied by taking into account the probability distribution of MSs’ location; however, a detailed mechanism of obtaining achievable transmission rate is not specified. In [11], an RS placement strategy with an advanced coding scheme was considered, and traffic demand was taken into account in determining the optimal RS location. In this scheme, a realistic user distribution is not considered. In [12], the optimal BS and RS locations was selected from among a candidate set of sites, and an integer programming technique was adopted in solving this problem. In this work, the tradeoff between load-balancing and throughput enhancement was not considered. In [13], the authors proposed a system capacity maximization scheme with multiple BSs and RSs for IEEE 802.16j networks, and experiments were conducted to confirm performance improvement through BS and RS placement in uniformly and non-uniformly distributed user scenarios. Several studies have assumed that the location of MSs in a cell follows a uniform distribution [11,14,15,16]; however, the distribution of MSs is greatly influenced by the existence of collective living infrastructure such as housing complexes, commercial districts, roads, large stadiums, etc. Therefore, the optimal RS location should be obtained considering unequal MS position distribution.

In this paper, we propose a method to find the sub-optimal location of RSs in a cell, based on the probability distribution of MSs’ location in a dual-hop relay system, where RSs operate in non-transparent mode. Specifically, the proposed scheme is constituted with the two stages of load-balancing and throughput enhancement. In the first stage, a rough position of RSs is determined by balancing overall traffic load in a cell site. In this stage, the computational complexity is very low, because the position of the RSs are adjusted by changing the distances and angles of RSs in a polar coordinate. In the second stage, the position of RSs are fined-tuned to enhance throughput, which results in the overall increase of spectral efficiency. This newly proposed two stage approach has an advantage in attaining the tradeoff between load-balancing and throughput enhancement. Moreover, in the load-balancing, unequal user distribution is considered for the purpose of reflecting a realistic user distribution scenario. The advantages of the proposed scheme are summarized as follows:

- RS positioning with low computational complexity considering load-balancing over a cell site. Instead of intriguing global optimizations, which adjust all the combinations of RSs’ position in a cell site, the proposed scheme can reduce the complexity by adjusting the distance and angular position of RSs in sector-by-sector manner.
- Reliable tradeoff between load-balancing and throughput enhancement through two stage approach. The fine-tuning of RSs’ position follows the first stage of load-balancing. Hence, the final result enhances the cell throughput with little sacrifice of load-balancing.
- Algorithm framework considers unequal user distributions to reflect realist user distribution scenarios. Instead of simple equal user distribution, a user distribution with clusters is taken into account.

The simulation studies show that the proposed scheme has fairly good performance.

## 2. System Model

### 2.1. Dual-Hop Relay Network

**Figure 1:**
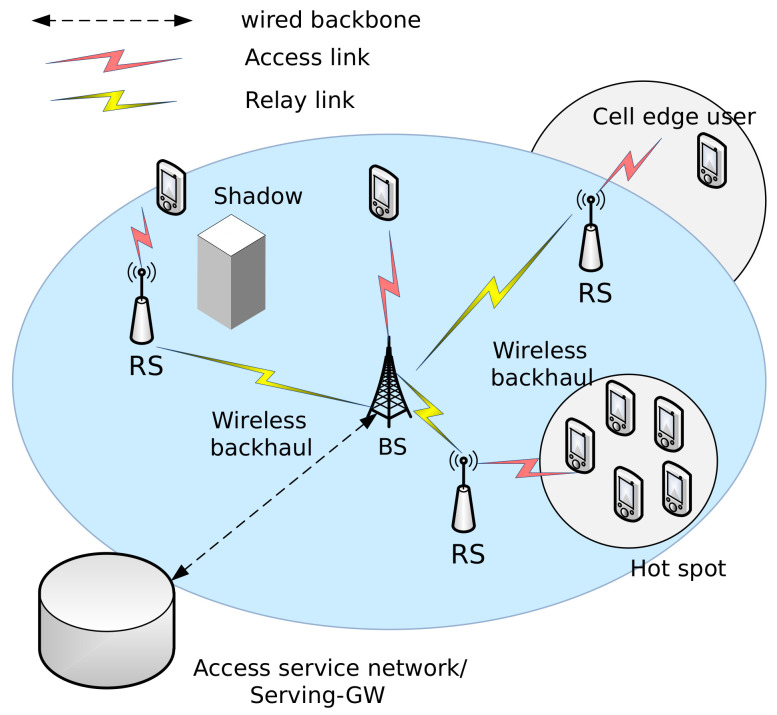
Dual-hop relay system.

Figure 1 shows dual-hop links in a relay system with three RSs operating in non-transparent mode. Some MSs are directly connected to a donor BS and the remaining MSs receive service via RSs. In this paper, a BS and RSs that serve MSs are referred to as service nodes.

**Figure 2:**
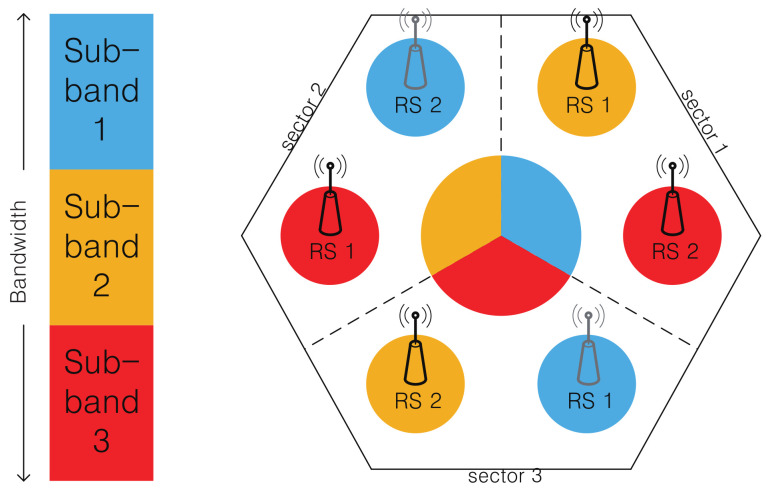
Orthogonal frequency allocation with two RSs per sector.

In a conventional cellular communication system, one cell is divided into three sectors, and each sector uses a different frequency band to reuse frequency while mitigating intracell interference [17]. Similarly, in this paper, one cell is divided into three sectors and the entire frequency band is divided into three sub-bands [18]. In addition, two RSs are deployed in each sector, constituting three service nodes per sector. Each MS located in a sector selects its own service node from among the service nodes of the sector. As we can see in Figure 2, we adopted the orthogonal frequency allocation scheme [18,19], which allocates sub-band to service nodes in a symmetrical manner. Specifically, a BS sector segment and two RSs in a sector use different sub-bands, while a sub-band allocated to an RS is reused by the other RS located at the opposite site. This allocation scheme reduces the interference among service nodes.

### 2.2. Resource Allocation for Dual-Hop Relay Network

**Figure 3:**
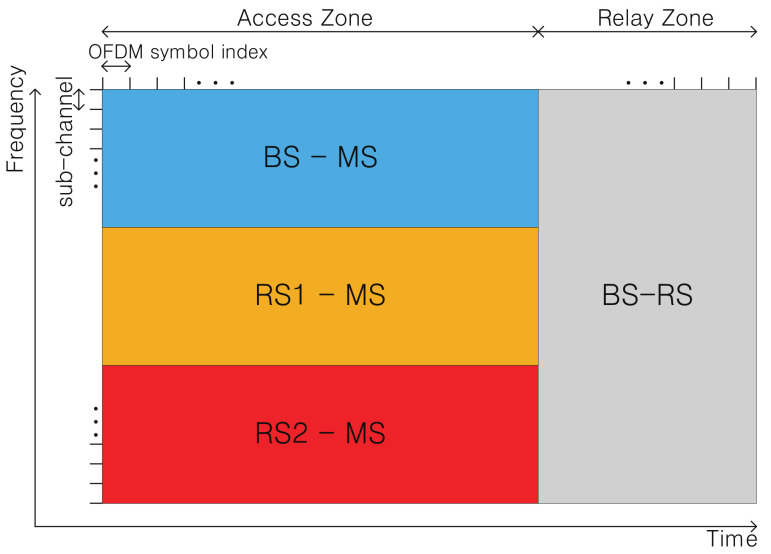
Downlink frame structure in a dual-hop relay network.

In a relay system, a frame is divided into two areas: an access zone (AZ) and a relay zone (RZ). AZ is a region of radio resource for the communication between MSs and service nodes; RZ is a region of radio resource for communication between RSs and BS [20]. In a dual-hop downlink (DL) connection, an RS receives packets from a donor BS through RZ, and then re-encodes and transmits this information to MSs through AZ. In this paper, we consider only DL connections. AZ and RZ are separated in an orthogonal frequency-division multiplexing (OFDM) symbols unit in a time domain, and AZ is divided into three sub-bands in frequency domain. Figure 3 shows a frame structure with partitioned resource areas for Sector 1.

As shown in Figure 2, there are three different service nodes in each sector (i.e., BS, RS 1, and RS 2). MSs in a sector select one of the service nodes to register. And the wireless channel between an MS and its associated service node can be modeled with the simplified path-loss model with log-normal shadowing [21] as follows:

(1) $$\begin{array}{c} P_{r}^{B/R,k}=P_{t}^{B/R}+K-10\gamma \log\frac{d}{d_{0}}-\psi_{dB}, \end{array}$$

where $P_{t}^{B/R}$ is the transmit power of the service node (i.e., BS or RS), *k* is MS index, $P_{r}^{B,k}$ is the received power of the *k*-th MS registered to BS, $P_{r}^{R,k}$ is the received power of the *k*-th MS registered to RS, *K* is path-loss factor, γ is path-loss exponent, *d* is the distance between MS *k* and service node, and $\psi_{db}$ is a Gaussian-distributed random variable with mean zero and variance $\sigma_{\psi_{dB}}^{2}$. Here, $d_{0}$ represents the reference distance between MS and service node in the above model. Initially an MS selects its service node by comparing the received SINRs from all the service nodes in the sector, and the SINRs of the service nodes in Sector 1 are represented as follows [22,23]:

(2) $$\begin{array}{ccc} & & \Gamma_{1}=\frac{P_{r}^{B,k}}{I_{1}+N},\mathrm{BS}\;\mathrm{to}\;\mathrm{MS}\;k\;\mathrm{link}, \end{array}$$

(3) $$\begin{array}{ccc} & & \Gamma_{2}=\frac{P_{r}^{R_{1},k}}{I_{2}+N},\mathrm{RS}\;1\;\;\mathrm{to}\;\mathrm{MS}\;k\;\mathrm{link}, \end{array}$$

(4) $$\begin{array}{ccc} & & \Gamma_{3}=\frac{P_{r}^{R_{2},k}}{I_{3}+N},\mathrm{RS}\;2\;\mathrm{to}\;\mathrm{MS}\;k\;\mathrm{link}, \end{array}$$

where for $\Gamma_{b}$ and $I_{b}$, b∈{1,2,3} is the index indicating BS, RS 1, and RS 2, respectively, $I_{b}$ is the sum of interference signals from other service nodes. Because a BS in a sector uses directional antenna with an angular range $120^{\circ }$, only part of the neighboring BSs act as interferers, but RSs use omni-directional antenna, and all the RSs, except for the currently selected RS, act as interferers. The MS selects a service node, $N^{\prime }$, which provides the highest SINR. This is expressed as follows:

(5) $$\begin{array}{c} N^{\prime }=\arg\max_{b}\left\{\Gamma_{b}\right\},\;\;b\in \left\{1,2,3\right\}. \end{array}$$

A dual-hop relay system may be affected by the distribution of MS locations. In an actual mobile communication system, the distribution of MS locations changes dynamically according to time and region. Therefore, if an RS is installed based on the instantaneous locations of MSs, performance degradation may occur when the locations of the MSs change. However, if an RS is installed based on the probability distribution of the MS locations, more stable performance will be guaranteed. However, a building MS location MAP from a real-world location data is a difficult task, because it is rather hard to obtain this data from a cellular operator and it often has a low-spatial resolution. Accordingly, we take an approach of synthesizing an imaginary sample MS location MAP. In order to make a MAP of the MS distribution for a cell, we divide the cell into many small spots and assume that the average number of MSs residing on each spot is available in a two-dimensional format. Specifically, $W_{n},n=1,2,...,N,$ is denoted as the average number of MSs residing on *n*-th spot, where *N* is the total number of spots over the cell area. This MAP can be generated in many different ways and this MAP generation is not a part of the algorithm to be proposed. The MAP generation method adopted in this paper is described in Appendix A.

## 3. Proposed RS Positioning Scheme

In this section, we propose a method of finding the sub-optimal location for an RS. The proposed scheme achieves load-balancing to reduce the risk of overloading. In addition, a fine adjustment of the RS position is conducted to enhance throughput. This method prevents overloading and improves the overall throughput.

### 3.1. RS Positioning for Load Balancing

Please note that all the service nodes in a sector have the same amount of AZ resource, as shown in Figure 2 and Figure 3. If RSs are deployed in locations where service nodes can serve a similar number of MSs, the risk of overloading is reduced. Default RS locations are arranged considering the range of the sector. Because there are six RSs in a cell, the location coordinate of the *r*-th RS in the *s*-th sector can be denoted as $L_{s,r},\;s=1,2,3,\;r=1,2$.

To adjust the location of each RS, the distance and the angle (degree) from the BS are required, and each position is expressed using the polar coordinate system as follows:

(6) $$\begin{array}{ccc} & & L_{s,1}=\left(d_{s,1},{\left(120s-90+a_{s,1}\right)}^{\circ }\right)\\ & & L_{s,2}=\left(d_{s,2},{\left(120s-90+a_{s,2}\right)}^{\circ }\right),\;s=1,2,3 \end{array}$$

**Figure 4:**
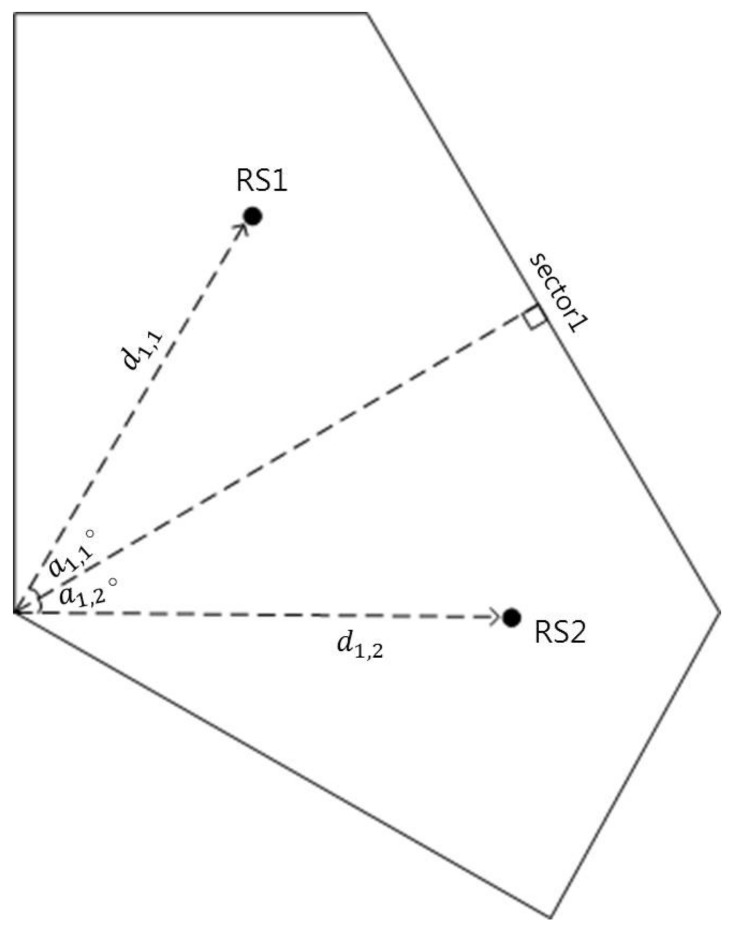
RS location using a polar coordinate.

Here, $d_{s,r}$ is a distance between the BS and each RS, $a_{s,r}$ is an angle from the reference angle. The reference angle is $30^{\circ }$ in Sector 1, $150^{\circ }$ in Sector 2, and $270^{\circ }$ in Sector 3. Figure 4 shows the location of RSs using polar coordinates in Sector 1.

To find the RS installation location, we use the newly generated MS location MAP. From $W_{n}$ of the generated MAP and the expected SINR at spot *n*, we can estimate the number of MSs to be registered to each service node. According to the service node determined for the MSs at spot *n*, the expected the number of MSs to be registered to each service node $W_{n}^{BS}$, $W_{n}^{RS1}$, and  $W_{n}^{RS2}$ are determined. For instance, if the service node of MSs at spot *n* is BS, $W_{n}^{BS}=W_{n}$, $W_{n}^{RS1}=0$, and $W_{n}^{RS2}=0$ are satisfied. Similarly, if the service node of MSs at spot *n* is RS1 or RS2, $W_{n}^{BS}=0$ and $W_{n}^{RS1}=W_{n},W_{n}^{RS2}=0$ or $W_{n}^{RS1}=0,W_{n}^{RS2}=W_{n}$ will be satisfied. Let $L_{s,r}^{\prime }=\left(d_{s,r}^{\prime },a_{s,r}^{\prime }\right)$ denote the new coordinate of an RS considering load-balancing among the service nodes. The process of obtaining $L^{\prime }$ is conducted in a sector-by-sector manner by updating the default coordinate *L*. First, the MS share for the BS (i.e., the ratio of the number of MSs registered to the BS to the total number of MSs in the sector) is adjusted, and this ratio can be expressed as follows:

(7) $$\begin{array}{c} S_{BS}=\frac{\sum_{n=1}^{N}W_{n}^{BS}}{\sum_{n=1}^{N}W_{n}}. \end{array}$$

In this step, $d_{s,1}^{\prime }$ and $d_{s,2}^{\prime }$ are adjusted so that $S_{BS}$ should reside in the range $\left[s_{l},s_{h}\right]$.

- If $S_{BS}<s_{l}$, $d_{s,1}^{\prime }$ and $d_{s,2}^{\prime }$ are increased by Δd, the RSs are moved away from the BS until $S_{BS}\geq s_{l}$ is satisfied.
  (8) $$\begin{array}{c} d_{s,1}^{\prime }\leftarrow d_{s,1}^{\prime }+\Delta d \end{array}$$
  (9) $$\begin{array}{c} d_{s,2}^{\prime }\leftarrow d_{s,2}^{\prime }+\Delta d \end{array}$$

When the RSs move away from the BS, a large number of the spots between the BS and the RSs become areas served by the BS, resulting in the increment of $S_{BS}$. At this step, if the distance from the RSs to the BS becomes too long, interference from the RSs with the neighboring cells becomes too severe. Hence, the maximum RS distance is defined, and if either $d_{s,1}^{\prime }$ or $d_{s,2}^{\prime }$ is to exceed this upper bound, the current step is terminated.

- In the case of $S_{BS}>s_{h}$, $d_{s,1}^{\prime }$ and $d_{s,2}$ are decreased by Δd, moving the RSs toward the BS until $S_{BS}\leq s_{h}$ is satisfied.
  (10) $$\begin{array}{c} d_{s,1}^{\prime }\leftarrow d_{s,1}^{\prime }-\Delta d \end{array}$$
  (11) $$\begin{array}{c} d_{s,2}^{\prime }\leftarrow d_{s,2}^{\prime }-\Delta d \end{array}$$

As the RSs approach the BS, $S_{BS}$ is decreased. Likewise, the minimum RS distance is set, and if either $d_{s,1}^{\prime }$ or $d_{s,2}^{\prime }$ is to exceed this lower bound, the current step is also terminated.

The next step is to adjust the MS shares of the RSs (i.e., the ratios of the number of the MSs registered to a specific RS, to the number of MSs registered to the RSs). The RS with the higher MS share is denoted as $RS_{H}$, and the other is denoted as $RS_{L}$. The MS shares for the two RSs are represented by $S_{RS}^{H}$ and $S_{RS}^{L}$, respectively, and these can be calculated as follows:

(12) $$\begin{array}{c} S_{RS}^{H}=\frac{\sum_{n}W_{n}^{RS_{H}}}{\sum_{n}W_{n}^{RS1}+\sum_{n}W_{n}^{RS2}}, \end{array}$$

(13) $$\begin{array}{c} S_{RS}^{L}=\frac{\sum_{n}W_{n}^{RS_{L}}}{\sum_{n}W_{n}^{RS1}+\sum_{n}W_{n}^{RS2}}. \end{array}$$

In this step, $a_{s,L}^{\prime }$, which is the angle of $RS_{L}$, is adjusted so that the $S_{RS}^{L}$ lies in the range $\left[r_{l},r_{h}\right]$, where $r_{h}=1-r_{l}$.

- When $S_{RS}^{L}<r_{l},a_{s,L}^{\prime }$ should be decreased by θ to move $RS_{L}$ toward $RS_{H}$ until $S_{RS}^{L}\geq r_{l}$ is satisfied.
  (14) $$\begin{array}{c} a_{s,L}^{\prime }\leftarrow a_{s,L}^{\prime }-\theta . \end{array}$$

Then, the service nodes of many spots located between $RS_{L}$ and $RS_{H}$ are changed from $RS_{H}$ to $RS_{L}$. At this time, if the two RSs are too close to each other, cell coverage cannot be efficiently extended. Therefore, the maximum angle needed to prevent this inefficient positioning is set. If  $S_{RS}^{L}\geq r_{l}$ is satisfied, the algorithm is terminated. However, if $S_{RS}^{L}\geq r_{l}$ is not satisfied even after changing $a_{s,L}^{\prime }$ to the maximum angle, we change the angle of $RS_{H}$ so that $S_{RS}^{H}\leq r_{h}$ is satisfied.

- To satisfy $S_{RS}^{H}\leq r_{h},a_{s,H}^{\prime }$ (the angle of $RS_{H}$), is increased by θ, moving $RS_{H}$ toward the boundary of the sector.
  (15) $$\begin{array}{c} a_{s,H}^{\prime }\leftarrow a_{s,H}^{\prime }+\theta . \end{array}$$

The maximum RS angle is also defined in the same way to prevent severe intracell interference between different sectors. If $S_{RS}^{H}\leq r_{h}$ is satisfied, the algorithm is terminated. The location of $RS_{L}$ is adjusted before $RS_{H}$ because $RS_{L}$ moves toward the center of the sector, while $RS_{H}$ moves toward the boundary of the sector. The latter has a higher risk of incurring severe intracell interference between two different sectors. If the $S_{RS}^{H}\leq r_{h}$ is not satisfied even after moving $RS_{L}$ and $RS_{H}$ to the maximum change angle, the distance of $RS_{L}$ is decreased by Δd and the angles of the RSs are initialized. Then, this algorithm goes back to the first step. The whole process is detailed in Algorithm 1.

### 3.2. Fine Adjustment of RS Position for Throughput Enhancement

The RSs located at $L^{\prime }$ can prevent overloading by adequately distributing the traffic load among the service nodes; however, additional fine adjustment of the RS locations is needed to increase the cell throughput. Cartesian coordinates are adopted in updating the RSs’ location to improve the cell throughput. $L_{s,r}^{\prime \prime }$ is the Cartesian coordinate representing $L_{s,r}^{\prime }$, obtained by Algorithm 1, and can be expressed as follows:

(16) $$\begin{array}{ccc} L_{s,r}^{\prime \prime } & = & \left(d_{s,r}^{\prime }\cdot \cos\left(a_{s,r}^{\prime }\right),d_{s,r}^{\prime }\cdot \sin\left(a_{s,r}^{\prime }\right)\right)\\ & = & (x_{s,r},y_{s,r}). \end{array}$$

**Algorithm 1** RS location for load balancing1:**Initialize:**2:   $L_{s,r}^{\prime }\leftarrow L_{s,r}$3:**Input:**4:   $s_{l},s_{h},d_{min},d_{max},\Delta d,\theta_{max},\theta ,r_{l},r_{h}$5:**step 1:** adjust BS share by changing RSs’ distance6:     **while ($S_{BS}<s_{l}$ and $d_{min}<d_{s,all}^{\prime }<d_{max}$)**7:       $d_{s,all}^{\prime }\leftarrow d_{s,all}^{\prime }-\Delta d$8:     **endwhile**9:     **while ($S_{BS}>s_{h}$ and $d_{min}<d_{s,all}^{\prime }<d_{max}$)**10:       $d_{s,all}^{\prime }\leftarrow d_{s,all}^{\prime }+\Delta d$11:     **endwhile**12:**step 2:** adjust RS share by changing RSs’ angle13:    **while ($S_{RS}^{L}<r_{l}$ and $a_{s,L}^{\prime }<\theta_{max}$)**14:      $a_{s,L}^{\prime }\leftarrow a_{s,L}^{\prime }-\theta$15:    **endwhile**16:    **while ($S_{RS}^{H}>r_{h}$ and $a_{s,H}^{\prime }<\theta_{max}$)**17:      $a_{s,H}^{\prime }\leftarrow a_{s,H}^{\prime }+\theta$18:    **endwhile**19:   **if ($S_{RS}^{H}<r_{h}$)**20:      $d_{s,L}^{\prime }\leftarrow d_{s,L}^{\prime }+\Delta d$21:      $a_{s,all}^{\prime }\leftarrow a_{s,all}$22:      **goto step 1:**23:   **endif**24:   **Output: $L_{s,r}^{\prime }$**

First, from the RSs’ position of Algorithm 1, the the total spectral efficiency of a sector is calculated. In this calculation, each sector should be divided by small spots, and the modulation and coding scheme (MCS)-levels and the average number of MSs for each spot should be taken into account. This is given by

(17) $$\begin{array}{c} K\left(L\right)=\sum_{n=1}^{N}\eta \left(\Gamma_{b(n)}\right)\cdot W_{n}^{b(n)},\;b\in \left\{1,2,3\right\} \end{array}$$

where *n* is a spot index, b(n) is the selected service node at spot *n*, $\eta (\Gamma_{b(n)})$ is the spectral efficiency at spot *n* with the SINR $\Gamma_{b(n)}$ from service node b(n), where spectral efficiency the number of bits can be transmitted over 1 Hz [24] and various spectral efficiency can be achieved by combining modulation schemes and channel coding rates. This can be obtained by referring to Table 1.

From the current RS position, an RS could move in eight directions with angles equally spaced by π/4. The candidate RS positions (including the current position) are denoted as $L_{j}^{T},j=0,1,...,8$, which can be expressed as:

(18) $$\begin{array}{c} L_{j}^{T}=\left\{\begin{array}{c} L_{s,r}^{\prime \prime },\;\;\;\;\;\;\;\;\;\;\;\;\;\;\;\;\;\;\;\;\;\;\;j=0\\ L_{s,r}^{\prime \prime }+x^{\prime }\cdot L_{d}\left(j\right),\;j=1,2,...,8, \end{array}\right. \end{array}$$

where $x^{\prime }$ is a unit distance of movement, $L_{d}\left(j\right)=\left(\cos\left(\pi /4\left(j-1\right)\right),\sin\left(\pi /4\left(j-1\right)\right)\right)$. For example, j=0 is adhering to the current position, j=π/4 is moving to upper right direction, j=π/2 is moving to upper direction, etc. The total spectral efficiency with respect to each candidate position is denoted as $K(L_{j}^{T}),j=0,1,...,8$. If $L_{j}^{T}$ is out of the bound of the sector, $K(L_{j}^{T})$ is set to 0. Now, we select a direction *j* that has the highest $K(L_{j}^{T})$ as follows:

(19) $$\begin{array}{c} j^{*}=\arg\max_{j}\left\{K\left(L_{j}^{T}\right)\right\},\;\;j=0,1,\ldots ,8 \end{array}$$

At this time, if $j^{*}$ is not 0, the RS position is updated as $L_{s,r}^{\prime \prime }\leftarrow L_{j^{*}}^{T}$, and these processes are repeated until $j^{*}$ becomes 0, because $j^{*}=0$ means that the performance will be degraded when the RS is moved further. Therefore, when $j^{*}=0$, this algorithm terminates and $L_{j}^{T}$ becomes the final RS installation location. The above procedure is detailed in Algorithm 2.
**Algorithm 2** RS location for throughput enhancement1:**Initialize:**2:   $j^{*}=1$3:**Input:**4:   $x^{\prime },L_{s,r}^{\prime \prime }$5:   **while ($S_{BS}<s_{l}$)**6:        $j^{*}=\arg\max_{j}\left\{K\left(L_{j}^{T}\right)\right\}$7:       if ($j^{*}\neq 0$)8:            $L_{j}^{T}=d_{s,all}^{\prime }+L_{s,r}^{\prime \prime }+\left(x^{\prime }\cos\left(\pi /4\left(j-1\right)\right),x^{\prime }\sin\left(\pi /4\left(j-1\right)\right)\right)$9:       **endif**10:   **endwhile**11:**Output: $L_{j}^{T}$**

The proposed algorithm requires the MAP to use the information of the average number of users on each spot; however, it is not dependent on the MAP generation method. Accordingly, the proposed algorithm is separated from the MAP generation.

## 4. Performance Evaluation

To analyze the performance of the proposed schemes, we performed a simulation of a dual-hop relay system. There were six cells around a center cell, and the sub-optimal positions for RSs in the center cell were obtained considering the interference from neighboring cells. The radius *R* of each cell was assumed to be 800 m. As shown in Figure 2, the cell was divided into three sectors, and each sector had two RSs. The main parameters considered in the simulation are shown in Table 2.

**Figure 5:**
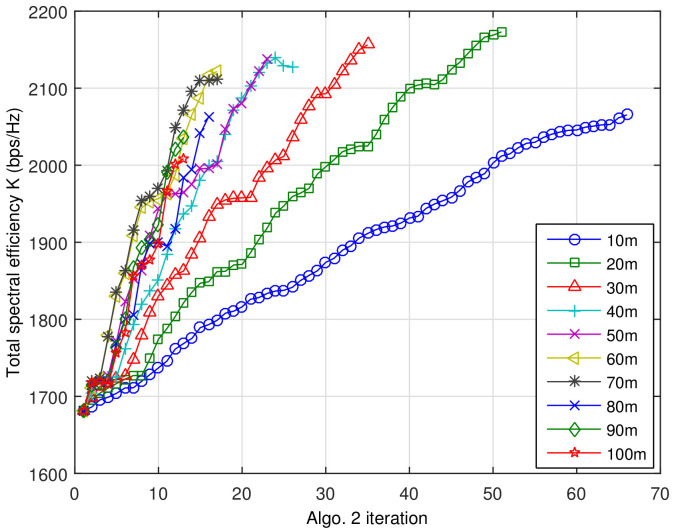
Spectral efficiencies with different step size $x^{\prime }$.

The proposed algorithms were run after determining appropriate input parameters. Preliminary tests were conducted to obtain these parameters, which are listed in Table 3. In determining RS moving step size $x^{\prime }$, the expected total spectral efficiencies with different step size $x^{\prime }$ is depicted in Figure 5.

In this figure, the last point of each line indicates the number of iterations before the termination of Algorithm 2 and the achieved spectral efficiency. As shown in this figure, when the step size is small, it takes many iterations before Algorithm 2 terminates by finding the sub-optimal spectral efficiency. On the contrary, if the step size is big, the total spectral efficiency increases sharply at early iterations; however, it fails to achieve the highest sub-optimal spectral efficiency. One important thing should be noticed is that the smallest step size 10 m fails to achieve the highest sup-optimal spectral efficiency. Since we adopt the unequal user distribution and log-normal shadowing, there are many local maximums. If the step size $x^{\prime }$ is too small, Algorithm 2 may stop at one of the local maxima. Other test cases showed that the unit distance of movement Δd and angle domain step size θ are rather insensitive to throughput performance, because Algorithm 1 focuses on load-balancing. Moreover, the ranges $\left[s_{l},s_{h}\right]$ and $\left[r_{l},r_{h}\right]$ also need to be carefully selected, because it affects the performance of Algorithm 1. In this paper, $\left[s_{l},s_{h}\right]$ and $\left[r_{l},r_{h}\right]$ were selected to achieve the lowest standard deviation of the number of MSs registered to the service nodes over the cell, considering the load-balancing capability of Algorithm 1.

**Figure 6:**
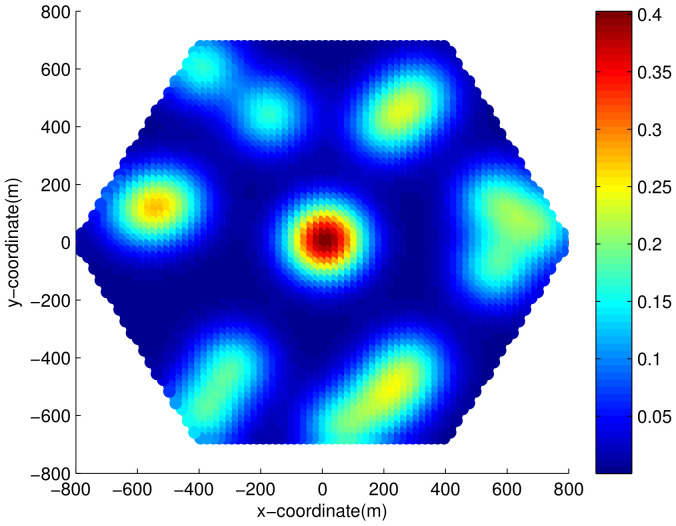
Generated sample MS location MAP.

Figure 6 shows $W_{n}$ in a generated sample MAP. As mentioned above, $W_{n}$ represents the expected number of MSs located at spot *n* and $\sum_{c=1}^{C}h_{c}$ is equivalent to the expected number of MSs in the cell. The number of dense areas *C* is 15, and the weight of dense area at the cell center is 50 and the remaining dense area is 20, so that an average of 330 MSs can be generated in the cell. The term $\sum_{n=1}^{N}W_{n}$ result is 296.71 on the generated MAP, which is smaller than 330 because the spots outside of the cell were excluded.

**Figure 7:**
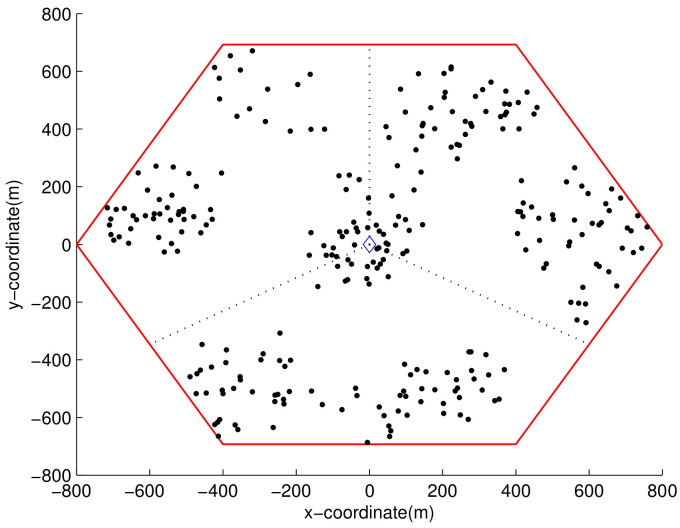
MS distribution sample drawn from MAP with user thinning.

The generated MAP cannot be directly used in simulation, because it describes the average number of users on each spot in a real value. Hence, the integer number of users for spot *n* over dense area *c* is acquired through Bernoulli trials with probability $w_{n,c}$ conducted $h_{c}$ times. Figure 7 shows a MS distribution sample, where user thinning is applied after being drawn from a generated MS location MAP. Before running the simulation, 10 different MS distribution samples were created with the sample MS location MAP. The default distance $d_{s,r}$ for RSs was set to 1/2 of the cell radius, and the default RS location angle $a_{s,r}$ was set to $30^{\circ }$. The simulation was performed for each distribution over 200 downlink frames.

**Figure 8:**
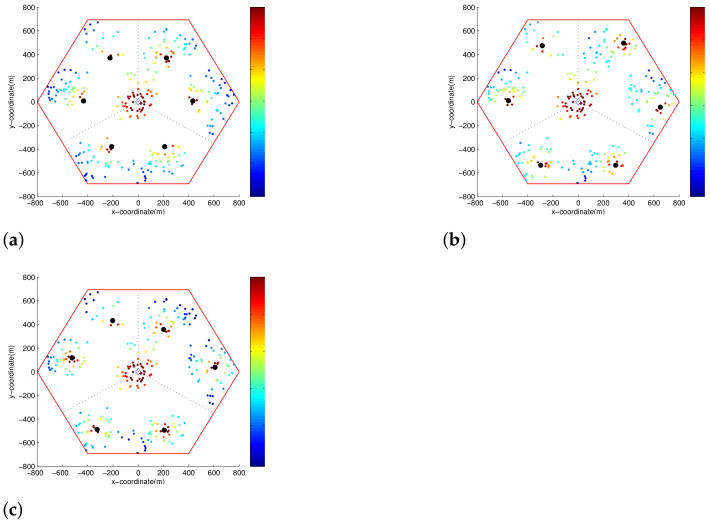
Variation of SINR distribution by RS location: (**a**) default position, (**b**) after Algorithm 1 and (**c**) after Algorithm 2.

In Figure 8 each color point represents an MS and its SINR, and the black points represent RSs. As we can see in this figure, the SINRs of the MSs change as the locations of the RSs change. Figure 8a is the SINR distribution with the default RSs locations, Figure 8b is the SINR distribution with the RSs positions obtained by Algorithm 1. In Figure 8b, compared to Figure 8a, since the RSs are moved toward the edge of the cell, the SINRs of the MSs located at cell edges were greatly improved by Algorithm 1. Meanwhile, the SINRs of the MSs located around default RS locations were somewhat decreased. In Figure 8c which represents the SINR distribution with the final RSs locations. As shown in Figure 8c, the SINRs of MSs were further improved by moving the RSs toward to the centers of the clusters. Hence, the enhanced throughput is expected in Figure 8c.

**Figure 9:**
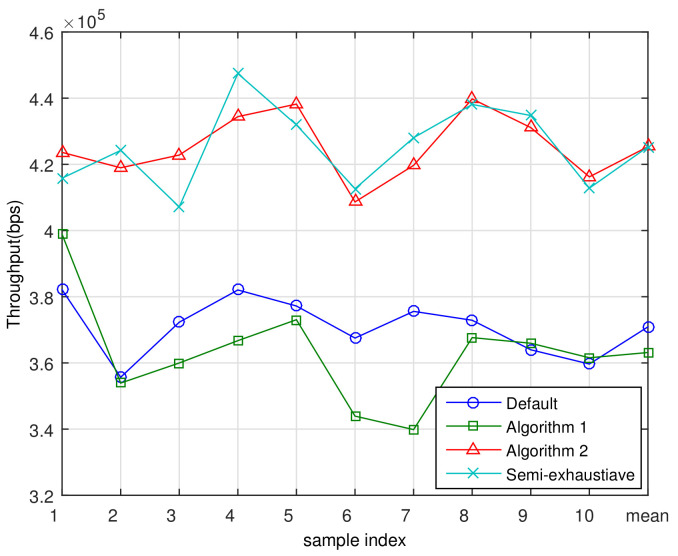
Throughput with different algorithms.

Figure 9 shows the cell throughputs for the ten samples of MS distribution and a mean throughput averaged over the ten samples. Please note that the purpose of Algorithm 1 is to balance the load among the service nodes and that the spectral efficiencies for the MSs are not considered in running Algorithm 1. Accordingly, the cell throughput may be reduced after Algorithm 1, because load-balancing may have some disadvantage in throughput maximization. After adjusting the RS positions using Algorithm 1, the average cell throughput decreased 8.1% compared to the default position. Using Algorithm 2, we found the sub-optimal RS location considering the expected number of MSs and their channel quality. By locating the RSs at the final positions obtained using Algorithm 2, the average cell throughput was increased by 16.4% compared to Algorithm 1, and increased by 14.9% compared to the default location. This confirms that Algorithm 2 greatly improves the average spectral efficiency, and that the purpose of Algorithm 2 was achieved. This improvement of spectral efficiency comes from the increment of SINR as clearly stated in Shannon capacity formula $C=\log_{2}\left(1+SINR\right)$ [26]. Accordingly, if the amount of bandwidth given in *B* Hz, the achievable throughput will be T=C·B.

In addition, for the purpose of proving the optimality of the proposed algorithm, a semi-exhaustive search-based sub-optimal scheme was compared with the proposed algorithm. Finding the sub-optimal RSs position through an exhaustive full search is an extremely difficult task. For instance, if 800 m cell radius and 10 m positioning resolution are assumed, each sector can have approximately 80×70=5600 different RS sites. If six RSs are in a cell, as in this paper, $2800^{6}≃4.819\times 10^{20}$ test cases should be examined, and it is computationally intractable. In the semi-exhaustive search, 35 m RS positioning resolution was adopted, and the sub-optimal RS position was searched per sector. This semi-exhaustive search and the proposed scheme achieve the similar performance with each other as shown in the Figure 9. However, note the computational complexity of semi-exhaustive search is still too high to be practically used.

**Figure 10:**
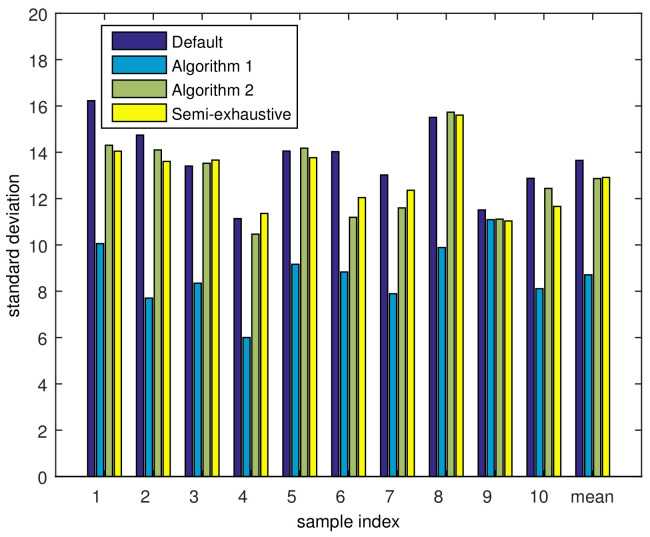
Standard deviations of MS distributions.

Finally, the simulation result evaluating the load-balancing capability of the proposed scheme is presented. Because the cell was divided into three sectors, there were nine service nodes in the cell. We evaluated the state of load-balancing through the standard deviation of the number of MSs registered to each service node. If all the service nodes have the similar number of MSs registered to oneself, the standard deviation taken over these numbers of MSs will be small; however, if some service node have many MSs, while others have small number of MSs, the standard deviation will be relatively large. Accordingly, standard deviation can be adopted as a metric representing the level of load-balancing. On average, there were 296.71 MSs on the generated MAP, and the actual number of MSs was reduced to 267.3 after user thinning. Accordingly, the average number of MSs per service node was 29.7. The standard deviation of each sample was calculated over nine service nodes, and the results are depicted in Figure 10.

As we can see in this figure, the overall standard deviation with the default RSs position is 13.7, which is a relatively high value. It means that some service nodes have too many MSs to serve while others have a small number of MSs to serve, which may incur a risk of performance degradation due to overload. However, Algorithm 1 decreased the standard deviation to 8.71, which is about 36.4% lower than for the default location, which makes the load more balanced. After running Algorithm 2, the standard deviation became 12.86, which is higher that Algorithm 1, but still lower than default position. Even though Algorithm 2 has increased the standard deviation by 4.15 compared to the result of Algorithm 1, it should be noted that the loss in the load-balancing is low while the throughput has increased greatly. As a result, we can improve both the load-balancing and the throughput by the proposed scheme, where an average of 6% standard deviation is decreased, and the throughput is increased by 14.9%.

## 5. Conclusions

In this paper, we propose a method of finding the sub-optimal RS location in a dual-hop relay system to reduce the risk of overloading and to improve the cell throughput. The proposed method was provided in the form of two stages. The first algorithm is a scheme for load-balancing and the second algorithm is a scheme for throughput enhancement. The simulation results after the first algorithm show that the standard deviation decreases because the MSs on overloaded service nodes are moved to other nodes. This can prevent performance degradation from overloading. We also confirmed that the total throughput increases after performing the second algorithm, which adjusts the RSs position to increase the total spectral efficiency. The proposed method is based on a sample MS location MAP assuming unequal MS distribution. The proposed method shows a good efficiency in finding a sub-optimal position with a low computational complexity, and it achieves a reasonable tradeoff between load-balancing and throughput enhancement, where the throughput is further enhanced after the load is balanced. In addition, the unequal user distributions reflecting realistic scenarios are taken into account. Therefore, the proposed algorithm shows enough benefit as a practical solution of determining the position of RSs in a cell site. The simulation results confirm that the proposed scheme shows excellent performance in selecting sub-optimal RS positions for dual-hop relay system.

## Figures and Tables

**Table 1:** MCS-levels accounting for received SINR [25].

Modulation	Coding Rate	SINR Threshold (dB)
QPSK	1/12	−3.14
QPSK	1/6	−0.73
QPSK	1/3	2.09
QPSK	1/2	4.75
QPSK	2/3	7.86
16-QAM	1/2	9.94
16-QAM	2/3	13.45
64-QAM	2/3	18.6
64-QAM	5/6	24.58

**Table 2:** Simulation parameters.

Parameter	Value
Number of cells	8
Number of sectors per cell	3
Number of RSs per sector	2
Cell radius	800 m
Tx power of BS	36 dBm
Tx power of RS	26 dBm
Constant path-loss factor (K)	−29 dBm
Channel bandwidth	10 MHz
Noise spectral density	−138 dBm/Hz
Number of subcarriers	720
Number of downlink symbols	29
Ratio of AZ:RZ	17:12
Carrier frequency	2 GHz
Frame length	5 ms
Number of spots in MAP	2288
Scheduling model	RR
Queue length in RS per MSs	6480 bit
Simulation frame number per MS distribution	200
Channel model	Simplified path-loss log-normal shadowing
Shadowing variance	8 dB

**Table 3:** Proposed algorithm input parameters.

Parameter		Value
BS share range	$s_{l}$	0.30
	$s_{h}$	0.35
RS share range	$r_{l}$	0.45
	$r_{h}$	0.55
RS distance limit	$d_{min}$	3R/16
	$d_{max}$	13R/16
Angle domain step size	θ	$1^{\circ }$
Maximum change angle	$\theta_{max}$	$5^{\circ }$
Unit distance of movement	Δd	20 m
	$x^{\prime }$	20 m
Default RSs location $L_{s,r}$	$d_{s,r}$	400 m
	$a_{s,r}$	30

## Data Availability

Data is contained within the article.

## Appendix A

First, a finite number of MS dense areas, such as group residences and large commercial buildings are arranged in a cell area. In arranging MS dense areas, one dense area is also located at the center of BS site in a cell [27], because, usually, a BS is in the center of a crowed area to service as many MSs as possible. Next, a weight factor is assigned to each dense area, where a weight factor is a value representing the number of MSs within the associated dense area. A standard deviation representing the dispersion of MSs from the center of that dense area is also assigned. Finally, the MS location distribution for each dense area is approximated using a two-dimensional Gaussian distribution function. In order to make a map of the MS distribution for the cell, we divide the cell into many small spots, and the probability of an MS (originating from a specific dense area) being located in a spot can be calculated by integrating the two-dimensional Gaussian distribution for the dense area, over the corresponding spot. This is given by

(A1) $$\begin{array}{c} w_{n,c}=\int_{A_{n}}\frac{1}{2\pi \delta_{c}^{2}}e^{-{\left(\frac{D_{n,c}}{2\delta }\right)}^{2}}dA,\left\{\begin{array}{c} n=0,1,...,N\\ c=1,2,...,C, \end{array}\right. \end{array}$$

where *n* is a spot index and *c* is a dense area index; $\delta_{c}$ is a standard deviation for MSs in dense area c; $D_{n,c}$ is the distance between the *n*-th spot and the *c*-th dense area; and $A_{n}$ is the size of *n*-th spot. *N* and *C* are the total number of spots and dense areas, respectively. Taking the total dense areas and their weight factors into account, the average number of MSs in a spot can be expressed as

(A2) $$\begin{array}{c} W_{n}=\sum_{c=1}^{C}h_{c}\cdot w_{n,c}, \end{array}$$

where $h_{c}$ is the weight of the dense area *c*. This process is not translating geographical data to MS distribution MAP but generating a plausible sample MS distribution MAP, which can be used as an input data to RS positioning algorithms. Based on $W_{n}$, the MAP describing the average number of MSs located in each spot can be generated. In obtaining a sample number of MSs for spot *n*, Bernoulli trials with probability $w_{n,c}$ should be conducted $h_{c}$ times and the results should be aggregated for each dense area c=1,2,⋯,C. After obtaining a randomized MS distribution sample from the MS distribution MAP, user thinning [28,29] is conducted. In the user thinning MSs with few neighboring MSs are excluded from the sample MS distribution, because, generally, users flock together rather than being independently located according to a single distribution function.
